# Approach via a small retroperitoneal anterior subcostal incision in the supine position for gasless laparoendoscopic single-port radical nephrectomy: initial experience of 42 patients

**DOI:** 10.1186/1471-2490-14-29

**Published:** 2014-04-04

**Authors:** Tatsuo Morita, Akira Fujisaki, Taro Kubo, Shinsuke Kurokawa

**Affiliations:** 1 Department of Urology, Jichi Medical University, Shimotsuke-city, Tochigi 3290498, Japan

**Keywords:** Retroperitoneal anterior subcostal incision, Supine position, Renal cell carcinoma, Radical nephrectomy, Laparoendoscopic single-port surgery, Gasless laparoendoscopic single-port surgery

## Abstract

**Background:**

Gasless laparoendoscopic single-port surgery (GasLESS) for radical nephrectomy (GasLESSRN) in the flank position is a minimally invasive treatment option for patients with T1–3 renal cell carcinoma (RCC). However, RCC patients considered suitable for supine positioning rather than flank positioning for radical nephrectomy are occasionally encountered. This study evaluated the safety and feasibility of approach via a small retroperitoneal anterior subcostal incision (RASI) in the supine position for GasLESSRN (RASI-GasLESSRN) on the basis of our initial experience.

**Methods:**

RASI-GasLESSRN was performed on 42 patients with RCC or suspected RCC from 2011–2013. The RASI, which was 6 cm long in principle, was made parallel to the tip of the rib from the lateral border of rectus abdominis muscle toward the flank in the supine position. The specimen was extracted via the RASI using a retrieval device. All procedures were performed retroperitoneally under flexible endoscopy with reusable instruments and without carbon dioxide insufflation or insertion of hands into the operative field.

**Results:**

RASI-GasLESSRN was successfully performed in all patients without complications. The mean incision length was 6.3 cm, mean operative time was 198 minutes, and mean blood loss was 284 mL. All 42 patients were classified as Clavien grade I. The mean times to oral feeding and walking were 1.1 and 2 days, respectively. The mean number of postoperative days required for patients to be dischargeable was 3.7 days.

**Conclusions:**

The approach via a small RASI in the supine position for GasLESSRN is a safe and feasible technique. RASI-GasLESSRN in the supine position is an alternative minimally invasive treatment option, especially for RCC patients considered suitable for supine positioning.

## Background

There are various approaches and options for radical nephrectomy (RN) for renal cell carcinoma (RCC): an open or laparoscopic approach; transperitoneally or retroperitoneally; via a single port, multiple ports, or a small incision; and with the patient in the supine position or flank position. However, the current RCC guidelines recommend the laparoscopic approach as the standard treatment or preferred option for T2 RCC patients [1,2]. Laparoendoscopic single-port surgery (LESS)-RN was recently developed. In LESS-RN, all instruments are inserted via a small incision with [3] or without carbon dioxide [4-6]. For LESS-RN without carbon dioxide, Kihara et al. [7] developed gasless laparoendoscopic single-port surgery RN (GasLESSRN), which was previously termed “portless endoscopic urologic surgery” (PLES) [5] or “minimum incision endoscopic surgery” (MIES) [6]. GasLESSRN is performed retroperitoneally in the flank position via a small incision 4–6 cm long under endoscopic magnification; it is performed in combination with stereovision and without carbon dioxide, trocar ports, or inserting the hands into the operative field. This procedure has been demonstrated to be a safe, feasible, cost-effective, and minimally invasive treatment option for T1–3 RCC with oncologic outcomes equivalent to those with conventional open radical nephrectomy [8]. However, GasLESSRN has several advantages: (1) the small incision as a single port, which permits the extraction of the kidney covered with Gerota’s fascia, is aesthetically advantageous; (2) carbon dioxide, which is a greenhouse gas associated with climate change [9] and confers a risk of pneumoperitoneum [10-12], is not required [13]; (3) the surgery uses reusable instruments, reducing waste and costs [14,15], which is particularly advantageous in developing countries. The cost-effectiveness, smaller environmental impact, and minimal invasiveness of GasLESSRN collectively warrant the pursuit of this technique. On the other hand, the positioning and type of incision are important initial steps for successful surgery without complications; they should be chosen according to the disease status, patient’s condition, and surgeon’s experience. We occasionally encounter RCC patients suitable for supine positioning rather than flank positioning for RN; these patients have conditions such as renal venous and/or arterial anomalies, lung diseases, and skeletal diseases or deformities as shown in living donor kidney harvesting [16]. Therefore, we adopted an approach via a small retroperitoneal anterior subcostal incision (RASI) in the supine position for RN, especially for GasLESSRN. Here, we present our initial experience and the details of an approach via a small RASI in the supine position for GasLESSRN, which is termed “RASI-GasLESSRN” herein.

## Methods

### Patients

A review of the medical records of Jichi Medical University Hospital revealed that from May 2011 to February 2013, RASI-GasLESSRN was performed on 42 patients with RCC or suspected RCC (Table 1). The present study was approved by the Institutional Review Board of Jichi Medical University, and informed consent was obtained from all patients.

**Table 1:** Patient characteristics and perioperative parameters

Number of patients	42
Age (years)	63 (46–79)^a^
Male/female	28/14
Laterality (right/left) 21/21	
Pathological examination	
Renal cell carcinoma	
pT1N0M0^b^	32
pT1N0M1	1
pT2N0M0	1
pT3N0M0	2
Angiomyolipoma	3
Renal hematoma	1
Hydronephrosis	2
Incision length (cm)	6.3 (6–8)
Operation time (min)	198 (113–342)
Blood loss (mL)	284 (30–670)
Days to oral feeding	1.1 (1–2)
Days to walking	2.0 (1–3)
Postoperative hospital stay (days)^c^	3.7 (3–5)

^a^Values are expressed as mean (range).

^b^pTNM pathological TNM stage.

^c^Postoperative days required for patients to be dischargeable, i.e., able to walk a long distance with full oral intake and without a drainage tube or analgesics.

### Surgical technique of RASI-GasLESSRN

**Figure 1:**
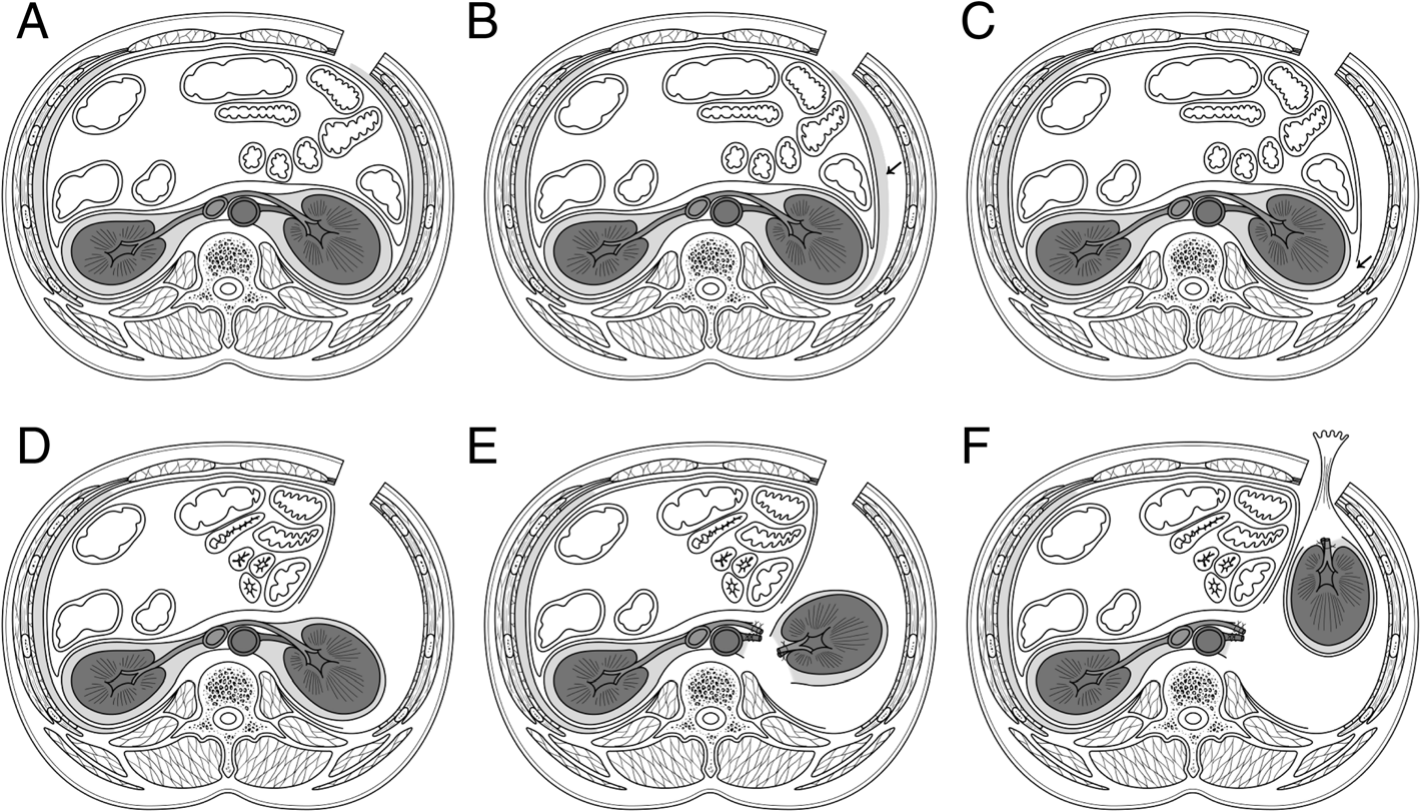
**Schematic presentation of GasLESSRN via RASI in the supine position. (A)** Subcostal skin incision. **(B)** Excision of fatty tissue (→) on the lateroconal fascia (LCF). **(C)** Incision of LCF (→) to expose Gerota’s fascia. **(D)** Medial retraction of peritoneum. **(E)** Dissection of renal vessels and freeing the remaining renal attachments. **(F)** Extraction of the specimen via RASI using a retrieval device.

**Figure 2:**
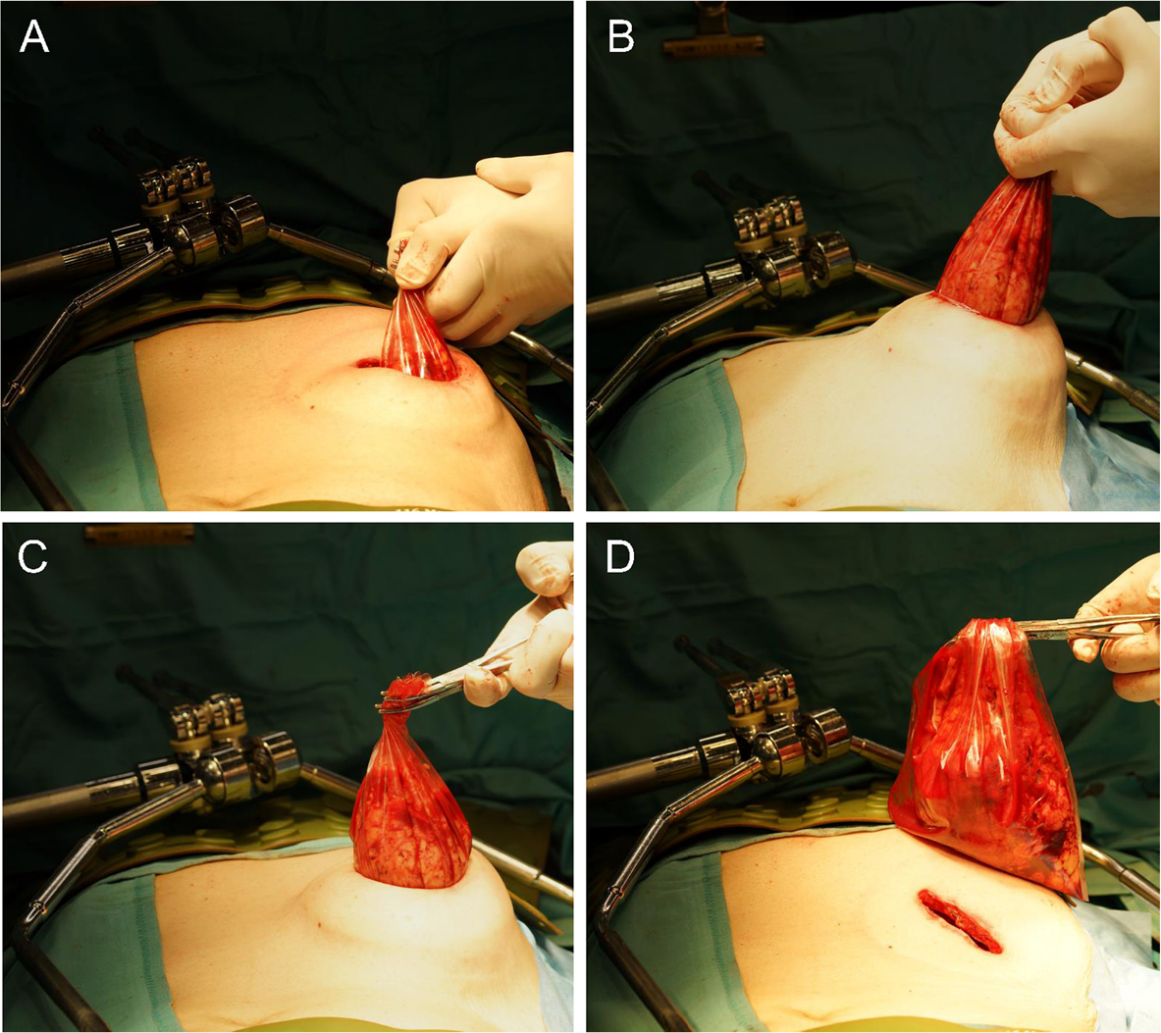
**Specimen extraction.** Images **(A)** before, **(B, C)** during, and **(D)** after specimen extraction via RASI using a retrieval device.

A schematic illustration of this procedure is shown in Figure 1. Under general anesthesia, the patient was placed in the supine position with back extension. A 16-Fr Foley catheter was indwelled, and a sequential compression device was placed on the lower extremities. A 6-cm subcostal incision, which varied depending on the size of the specimen to be extracted, was made from the lateral border of rectus abdominis muscle toward the flank parallel to the tip of the rib without cutting the rectus abdominis muscle. Only the external oblique muscle was incised in the incision line, and the internal oblique and transverse muscles were split to avoid injuring the subcostal nerve without rib Resection. A surgical retractor (Gray Surgical Retractor System, Gray Surgical, Australia) was set, and subsequent procedures were performed using long instruments under direct vision and video imaging with a flexible endoscope. The lateroconal fascia (LCF) was exposed by excising surrounding fatty tissue (i.e., flank pads), and the landmark of the quadratus lumborum muscle was identified. The LCF was then incised longitudinally on the lateral or posterior side of the kidney, exposing Gerota’s fascia. The peritoneum was retracted medially with blunt dissection to expose the anterior surface of the kidney covered with Gerota’s fascia. A wound retractor (Alexis Wound Protector/Retractor™, Applied Medical Resources Corp., USA) was set to obtain an adequate operative field. After dissecting toward the renal hilum, the renal artery and vein were exposed, double-ligated, and transected. For the left kidney, the adrenal, lumbar, and gonadal veins were ligated and transected. The ureter was isolated, ligated, and transected at the level of the lower pole of the kidney. After anterior, posterior, and medial dissection along Gerota’s fascia, the cranial border of Gerota’s fascia was dissected. Ipsilateral adrenalectomy was performed en bloc when necessary. After freeing the remaining renal attachments, the specimen was extracted via the RASI using a retrieval device (Kobamed Flexible Catcher™, Kobayashi Medical Co., Ltd., Japan) (Figure 2). Lymphadenectomy was performed when necessary. Hemostasis was carefully ensured. The dorsal spine extension was released after placing a 16-Fr drainage tube. All 3 muscle layers including the transverse abdominis, and internal and external oblique muscles were approximated using 1–0 monofilament polyglycolate sutures. The wound was subsequently washed with physiological saline. The skin was closed intracutaneously and covered with hydrocolloidal dressing (Karayahesive™, ALCARE Co., Ltd., Japan). All procedures were performed without gas insufflation or inserting the hands into the operative field.

## Results

The perioperative data are summarized in Table 1. RASI-GasLESSRN was successfully completed in all 42 patients without converting to conventional open RN. The mean operative time was 198 minutes, and mean estimated blood loss was 284 mL. All specimens were retrieved successfully via the RASI, which had a mean length of 6.3 cm. Pathological examination revealed RCC in 36 patients and benign renal diseases in 6 patients including 3, 1, and 2 with angiomyolipoma, renal hematoma, and hydronephrosis, respectively (Table 1). No blood transfusions were performed. All 42 patients were classified as Clavien grade I. The postoperative course was uneventful in all patients. The mean times to oral feeding and walking were 1.1 and 2 days, respectively. To evaluate hospital stay, we used the number of postoperative days required before patients were dischargeable, i.e., the ability to walk a long distance with full oral intake and without a drainage tube or analgesics [5]. In Japan, patients, particularly elderly patients, frequently choose to remain in the hospital for as long as possible after surgery, because the public medical insurance system covers most of the costs. Therefore, the mean number of postoperative days before patients were dischargeable was 3.7 days. All 36 RCC patients remained well without any evidence of recurrence at a mean follow-up of 22 months (range: 10–30 months).

## Discussion

This study presents our initial experience with RASI-GasLESSRN in the supine position. The principle surgical technique of GasLESSRN implemented in the present study is similar to that of Kihara et al. [4-6], except the approach via a small RASI in the supine position. Among the 80 cases of GasLESSRN in the flank position recently reported by Kihara et al. [5], the mean incision length was 6.6 cm, mean operative time was 3.1 h, and mean estimated blood loss was 324 mL. In that series, complications included pleural injury and hemorrhage from the vena cava—both of which were repaired intraoperatively by suturing. Blood transfusion was performed in 3 patients (3.8%). The mean numbers of days to oral feeding, long walking (>100 m), and possible minimal hospital stay were 1.4, 1.4, and 4.8 days, respectively. The present results (Table 1) are comparable to those of Kihara et al. [5], suggesting the approach via a small RASI in the supine position is a safe and feasible technique for GasLESSRN.

The approach via a small RASI in the supine position presented herein has several advantages. First, there is no delay due to the positional change from the supine to flank position. Second, supine positioning is helpful for RCC patients with skeletal diseases or deformities such as contralateral shoulder joint diseases, nonunion of the clavicle, and scoliosis [17]; it also avoids the potential risks associated with flank positioning, including peripheral nerve injuries such as brachial plexus neuropraxia [18], rhabdomyolysis [19,20], unilateral pulmonary edema (i.e., down lung syndrome) [21], and atelectasis in the dependent lung [22,23]. Connor et al. [16] compared the anterior extraperitoneal approach to the flank approach for living donor open nephrectomy in a series of 36 familial donors; they conclude the former procedure confers not only superior visualization of the renal vessels, but also superior projected benefits for donors with obesity, age >45 years, pulmonary disease, multiple renal arteries, and skeletal deformities. Third, this anterior approach provides better visualization of the renal hilus, adrenal gland, and anterior surface of the kidney. In living donor kidney harvesting, a retroperitoneal anterior subcostal incision [16,24,25] or pararectal incision [26] in the supine position provides excellent access to the renal vessels without serious complications. However, the most commonly used surgical approach for living donor kidney harvesting is performed in the flank position [27]. Furthermore, this advantage suggests RASI in the supine position can be applied to nephron-sparing surgery, which is currently the preferred option for T1 RCC [1,2], especially for RCCs located at the upper pole, anterior surface, or renal hilus. Finally, this anterior approach also provides a familiar anatomical view of the kidney, especially for surgeons familiar with conventional RN via a transperitoneal anterior subcostal incision (TASI) in the supine position [28]. Furthermore, it is easy to convert the approach into the conventional TASI with the incision of the peritoneum if required, although no such conversions were performed in the present series.

Despite its many advantages, the approach via RASI in the supine position has some limitations that should be mentioned. First, retracting the peritoneum medially and exposing the anterior side of the kidney covered with Gerota’s fascia is occasionally time consuming; this is because the site of the lateral peritoneum reflection is located anterior to the posterior axillary line. Second, regarding aesthetics, the subcostal incision, which is not along Langer’s lines, might result in the formation of an unsightly scar in the upper quadrant of the abdomen; meanwhile, a skin incision along Langer’s lines heals with an almost invisible hairline scar [29]. Furthermore, the present study has some limitations including its retrospective nature, the small number of patients, a lack of comparison with other minimally invasive RNs, and the short follow-up period. Although the present study demonstrates the feasibility and safety of the technique of RASI-GasLESSRN in the supine position, further study is required to confirm the present results in RCC patients considered suitable for supine positioning rather than flank positioning.

## Conclusions

The results of the present study show for the first time that the approach via a small RASI in the supine position for GasLESSRN is a safe and feasible technique. Despite some drawbacks and limitations, RASI-GasLESSRN in the supine position is an alternative minimally invasive treatment option, especially for RCC patients considered suitable for supine positioning.

## Abbreviations

RASI: Retroperitoneal anterior subcostal incision; RASI-GasLESSRN: Gasless laparoendoscopic single-port radical nephrectomy; RCC: Renal cell carcinoma; RN: Radical nephrectomy; LESS: Laparoendoscopic single-port surgery; LCF: Lateroconal fascia; MIES: Minimum incision endoscopic surgery; PLES: Portless endoscopic urologic surgery; TASI: Transperitoneal anterior subcostal incision.

## Competing interest

The authors declare that they have no competing interests.

## Authors’ contributions

TM performed data collection and analysis, and drafted the manuscript. TM, AF, TK, and SK performed the operations. All authors have read and approved the final manuscript.

## Pre-publication history

The pre-publication history for this paper can be accessed here:

http://www.biomedcentral.com/1471-2490/14/29/prepub
